# Hydrate of Chloral

**Published:** 1871-08

**Authors:** T. S. Powell

**Affiliations:** Atlanta, Ga.


THE GEORGIA

### MEDICAL COMPANION:

A MONTHLY ADVISER.

VOL. I. ATLANTA, GA., AUGUST, 1871. NO. 8

### PART I.

### Original Communications and Special Selections.

### HYDRATE OF CHLORAL.

BY T. S. POWELL, M. D., ATLANTA, GA.

No remedy presented to the notice of the medical profession within the past few years has attracted so much attention as Chloral. We have read the reports of experiments with it with deep interest, and with hope we await further developements.

It is still upon trial, and while it is likely to become a "fashionable remedy," prescribed without due discrimination in the hands of a certain class of men given to hobby riding, on the one hand, and attacked with a little thought on the other hand, by another class who claim to be conservative, yet the reports of those who have given the remedy a thorough trial, are to a certain extent of such uniform character, as to lead us to hope that it will soon take its proper position among the important remedies of the Pharmacopœia—its merits properly appreciated.

Chloral was discovered by Baron Liebig in the year 1832, but it remained simply a chemical curiosity until Liebreich, of Berlin, suggested it as an hynotic and anæsthetic remedy, as late, we believe, as 1869.

We have seen little said on the theoretical question of its mode of action. The theory of Liebreich, we believe, is that the effects of Chloral Hydrate are produced by chloroform, evolved by the decomposition of chloral by the alkalies of the blood ; in other words, it is the mild but continued action of chloroform. This theory will perhaps account for the most of the effects of chloral.

Yet it is now denied by high authority, that it has any anæsthetic properties at all. Is it possible, then, that the anæsthetic effects of chloroform are dependent upon the rapidity of its introduction into the system? But leaving this question, we subjoin extracts from various sources, only premising that we do not copy the various articles entire, but generally the summary or conclusions of the writer.

The editor of the London Lancet says of chloral hydrate "In the first place the term "anæsthetic" should no longer be applied to chloral, for it has entirely failed to make good its claim to this reputation; even the largest doses do but produce a heavy and prolonged sleep, which is, however, essentially different from true anæsthesia. On the other hand, as a producer of sleep, chloral is in many respects unrivalled, for though, like every other remedy, it fails in a considerable number of cases, it does succeod in a very large number. In fact it is inferior, in certainty, as a hypnotic, to opium alone. Moreover, it is greatly superior to opium, and almost every other drug, in the character of its sleep-producing action; there are no attendant symptoms of cerebral oppression; the sleep, though often prolonged, is light and refreshing, and no unpleasant after-symptoms are experienced. It is important to observe, however, that this description only applies to the use of moderate quantities, and that not only unpleasant, but highly dangerous symptoms have been produced by over-doses, which, we regret to see, are very commonly used. Very careful inquiry leads us to assert that it is both unnecessary and dangerous to give larger doses than twenty to thirty grains, repeated once or twice, if necessary, for hypnotic purposes. Doubtless it might happen that a consumptive patient might take much larger doses with impunity, but the one hundredth might present the alarming symptoms described by Dr. Reynolds, in a recent number of the Practitioner, as produced by a dose of fifty grains, and these symptoms might take a fatal turn. As a remedy for pain, chloral holds a very varying place in the estimation of medical men—some rating it highly, and others thinking it almost worthless. Perhaps the safest estimate of its powers over pain is that it only exerts an indirect influence, by inducing a disposition to sleep, in which the pain is forgotten. Certainly it has entirely failed, in the hands of the present writer, to relieve pain of a pure neuralgic type. On the other hand, there is a good deal of evidence that it relieves suffering where the parts are very tense and where mere arterial throbbing counts for much in the production of pain. Thus it has been favorably spoken of for its effects upon gout. And this fact, if it be correct, corresponds with certain observations which have been made as to its action on the circulation. Both from sphymographic experiments on healthy persons and on patients, and also from the details of the nearly fatal case reported by Dr. Reynolds, there is reason to think that chloral exerts a contracting influence upon the arterials powerful in proportion to the dose, and it may well be that arterial throbbing is checked by this kind of influence on the whole. However, there can be little doubt that the great function of chloral is that of hypnotic, and calmer of general nervous irritability. In delirium tremens it is excellent, and it is probable that with two such weapons for choice as bromide of potassium and chloral we shall be able almost entirely to dispense with the use of opium, which is so uncertain and dangerous a remedy in that disease. In the state of sleeplessness which threatens the access puerperal mania, chloral is probably an unequalled remedy. In melancholia, its action as a hypnotic appears to be powerful and remarkably sure. In mania, also, it acts well enough as a hypnotic, though there seems some division of opinion as to whether it does permanent good. We may also state that in the irritable condition of aged persons, who find it difficult to sleep for any length of time continuously, a single dose of thirty grains of chloral appears to answer excellently well. The minor uses of the drug in relieving more trivial conditions of nervous irritation and in alleviating painful spasmodic symptoms of various kind, are probably considerable."

We have given the above article from the Lancet entire, as it is perhaps the most correct "criticism," so to speak, we have met with on the subject, and contains in a condensed form, what we find in many others. That it is a valuable medicine we have no doubt, but that great harm has and will continue to result from the too free use of it, is equally clear to our mind, and we would advise great caution in its use.

The late Sir J. Y. Simpson, Bart, M. D. C. L., in the Medical Times and Gazette, of January 1st, 1870, says: Hitherto I have employed it principally as an hypnotic and anodyne. In sufficient doses I have found it, as a general law, as sure a producer of sleep and soother of pain as opium or any of its preparations. It is usually swifter in the induction of its narcotism, more tranquil in its action, and more prolonged in its effects, than opiates are when taken as hypnotics; but above all, it seems in a great measure free from some of the minor drawbacks and disagreeable accompaniments produced by a full and large dose of opium.

In this respect it appears to me to fulfill successfully the indications which I predicted in the extract above given," (referring to an extract from an article published several years before) "of being a narcotic as powerful, and indeed more powerful, than opium, and yet, without either its direct constipating effects, or its indirect tendency to excite subsequent nausea, vomiting, &c. The sleep induced by a full dose of it, steals on without any premonitory symptoms. It is usually .deeper, and yet more quiet and calm, than that produced by opium, and it does not leave subsequently the thirst, dry throat and tongue, disturbance of stomach and appetite, and languor of mind as well as body, which most persons unaccustomed to the use of opium, commonly feel after a deep and narcotic dose of that drug. * * * In the present remarks I have spoken specially of the somniferous and hypnotic powers of chloral. I have used it for other purposes, but it is not my intention to speak of them at present. It will not fulfill all the many and almost endless indications for which opium is used in medicine, but I have seen enough to convince me that it will prove a very valuable anodyne in some cases of neuralgia, hysteralgia, dysmenorrhœa, pleurodynia, &c., and in the pains attendant upon cancer and acute local inflammations. In several instances it has seemed to me, when given in small and repeated doses, to soothe down both acute and chronic coughs, with remarkable effect, and I have known it to relieve asthma."

One of the most important advantages we have in the use of chloral, is its adaptation to many cases where opium is contraindicated on account of the tendency in the latter drug to disturb the functions of digestion, and hinder all the eliminating processes of the body. It is claimed to be specially useful in febrile diseases with cerebral complications. On this point, Dr. James B. Russell B. A. Physician to the city of Glasgow Fever Hospital, in a communication to the Glasgow Medical Journal, says: "In the treatment of fever, one has necessarily an unusual opportunity of studying the effects of hypnotics and sedatives. Very few cases pass through the physician's hands without at some period demanding the use of some means of producing sleep artificially; and the question which to employ is neither unimportant nor of easy solution. I have a very decided opinion as to the danger attending the use of opiates in fever, no matter how guarded with such elements as antimony, gray powder, &c. Nothing astonishes me more, as being opposed to my whole experience, of their disastrous results, than the free and unguarded way in which preparations of opium, either alone or combined, are recommended even in treatises on fever, and employed by some practitioners. From the want of a better agent, I have hitherto, after Graves, used Battley with tartar-emetic (5 drops to ½ grains,) but I never order such a draught without apprehension, and have repeatedly seen patients put into a dangerous state of narcosis even by one such minute dose—a state always requiring active and prompt measures to save the life, and not unfrequently ending in fatal coma or pulmonary engagement, or congestion."

After discussing further the danger attending the use of opium, and comparing its effects with those of chloral, he says, "I am well aware that the chemical experiments here recorded are but limited, and must be subjected to prolonged trial and observation before they can be thoroughly established. Still I see in chloral sleep something different from opium sleep, and both positive and negative properties in the former drug, which make it safer than the latter.

1. The action of the pupil, to which I have already alluded, illustrates excellently the close approximation to natural sleep which chloral bestows, and the difference between it and opium in physiological effect. In all forms of sleep the pupil is contracted to the utmost. But in natural sleep, the moment the sleeper is roused and the eyes are opened, the pupil expands; while in an opium sleep, the pupil remains contracted. it may be only for a few seconds, or for minutes or hours. This illustrates, in little, the general condition of the nervous and muscular system, and other narcotic forms of sleep. As we have seen, the chloral sleeper may be roused at any time. He is at once in the full command of his faculties. He may take food, pass urine, cough with full strength, expectorate, &c., and whenever the temporary excitation ceases, he may drop off into unconsciousness again. Indeed, as a rule, the chloral sleep consists of a continuous initiatory stage, followed by a period which may extend over six, eight or more hours of sleep, interrupted by intervals, in which food, &c., may be given without the necessity of waking, or any risk of re-establishing the excitement. Not so with a patient after an opiate, which will produce an equally sedative effect. His pupils are small; if his lungs are gorged with mucus, he has neither the intelligence to see the necessity, nor the muscular energy at command to cough and expectorate.

2. The excretions are not affected by chloral. They are by opium. The bowels are made costive, the urine is scanty, the mouth parched, the skin dry.

3. The administration of an opiate to a child is, in any circumstances, a procedure which the practitioner adopts with misgivings. Chloral may be given with perfect success.

4. Opiates, even as combined by Graves with tartar-emetic, are uncertain and very frequently futile.

As already stated, I have met with no patient who resisted the action of chloral."

We find many cases reported in which chloral has been used for the relief of various nervous affections. We notice one case of traumatic tetanus in which recovery occurred, from one and a half to three drachms were given daily, complete recovery was established in one month. Trismus has been successfully treated with the same remedy. Much benefit has been realized in the treatment of whooping-cough, by various practitioners. Chorea has been successfnlly treated; a friend of the writer used it in a severe case of chorea. In this case the spasm would last several days with such severity as to render the patient unable to speak or swallow. A dose of thirty grains never failed to secure relaxation and sleep for from five to seven hours.

Chloral has been found very useful as a hypnotic in the treatment of the insane. Dr. Tuke, Medical Sup't. of the Fife and Kinros Lunatic Asylum, used it with benefit in a number of cases. He says, "I believe it to be the most valuable means of procuring sleep which has yet been introduced into the pharmacopœia of the asylum physician. The only difficulty is to ascertain the exact dose for each case; but this is obviated by beginning with half drachm doses and increasing them by ten grains until the l mit is found.

An interesting discussion relative to chloral was had at the last meeting of the Association of medical superintendents of American Institutions for the Insane. We find the proceedings of the Association in the American Journal of Insanity, from which we make a few extracts, not, however, giving the remarks of the different speakers in full. Dr. Kirkbride said, "The results of our experience may be briefly stated to be, that with many cases it acts promptly, producing sleep which lasts uninterruptedly for several hours, and without any unpleasant after-effects; that with others it acts less decidedly—the sleep being light, and the patient easily aroused; in some the effect is unfavorable, producing excitement, a kind of intoxication rather than sleep. It has done quite as much as we expected from it. Like the bromide of potassium, it is an adjunct to morphia, but in no way a substitute for it, and to me it has seemed to lose its effects much sooner. I have the experience of some patients not insane, and these have varied very much. In one it was only excitement and wakefulness that followed its use, and this condition continued till a full dose of morphine was taken. In another in twenty-grain doses, sleep was induced; but the gentleman, a person of great intelligence fancied that, although apparently asleep, he knew what was going on around him.

We have often used it among a set of habitually noisy patients, and very often with the result of having a quiet ward for the night, but we cannot depend upon it, if long continued, for such patients. Patients sometimes go to sleep soundly in ten or fifteen minutes, but the effect is not generally so rapid or permanent as is occasionally reported in private practice; a lady, for instance, after taking twenty grains went to her glass to arrange her hair, but the overpowering disposition to sleep that came over her compelled her to desist; and, lying down, she slept on continuously till next morning. I have seen nothing like this. One of my assistants found, after doses of thirty grains, merely, the first symptoms of intoxication."

Dr. Walker considers chloral very useful, especially among elderly people who sleep with difficulty, and who come out greatly comforted and refreshed during the day, by its use. Generally, in doses of fifteen grains, they obtain a good night's rest, and seem to be comfortable throughout the following day."

Dr. Gray says, "we have used chloral from twenty to forty grains, and in some instances, sixty grains, and often in forty grain doses repeated." We have prescribed it in acute mania, with the variable success spoken of by Dr. Kirksbride. In some cases it seemed to have little or no influence at one time, and a few days afterwards, in the same cases, a decided effect. We have had good results from its administration in noisy, chronic, and paroxysmal mania, and in sleeplessness and melancholia, also in cases of rheumatism with pain, and sleeplessness at night. We have used it in a variety of conditions as a hypnotic, but we have not found much benefit from it in doses of less than thirty grains. Our observation corresponds with that of Sir James Y. Simpson, that as a hypnotic, it should be given in doses of not less than 40 grains. He frequently gave it in fifty or sixty grain doses.

The question has been asked in this discussion whether or not it allays pain. I think it does in many cases by preventing or controlling spasm. I have prescribed it in a number of cases of painful menstruation, with happy effect, in doses of twenty to forty grains. Also in several cases of severe colic, in doses of 20 to 40 grains. Also in several cases of severe colic, in doses of twenty grains, combined with a drachm of paregoric, given at intervals of two to four hours.

For two cases of threatened abortion, twenty grains of chloral and a drachm of paregoric acted admirably, given every four hours.

For neuralgia, where morphia has not been tolerated, even by hypodermic injection, I have found chloral produce comfortable and refreshing sleep.

In instances where its administration was followed in the morning by depression, a cup of strong coffee afforded relief. In one case of neuralgia, not an insane person, where the pain was so intolerable as not only to prevent sleep, but to cause a resolute woman to cry out with pain, chloral secured sound sleep. In this case hypodermic injections of morphia allayed the pain, but induced wakefulness, and produced subsequent nausea and depression. The nausea and depression were entirely relieved, although the injections were continued by the administration of twenty grains of chloral, twice a day, and sleep was secured by forty grains of chloral at night. In the cases above referred to, and in two of Bright's diseases, I have thought chloral produced an influence in relieving pain. I have no doubt that it is a most valuable remedy, and a great boon to the profession and humanity; and while we are not to expect that it is going to do away with the use of any other hypnotic, for it requires the entire resources of medicine for the physician to meet all disordered conditions, yet I think it may be placed on the list as one of the most useful hynotics."

We would be glad to give further extracts from this discussion; however, the experience of the other gentlemen does not differ very materially from that above given.

The dose of chloral, as generally used, seems to vary from fifteen to forty, or even sixty grains. The dose necessary to produce a given effect varies considerably in different persons, and in the same individuals, under different circumstances, just as we often see in almost every remedy. A friend reports one case in which fifteen grains produced fourteen hours' sleep. The same case had repeatedly taken the same amount without producing any such decided effect. Our experience in the use of chloral has been limited compared with the gentlemen whose opinions we have given, and while we fully appreciate their opinions and hope our readers will be greatly benefitted by their experience, we would advise the greatest caution and discretion in its use, especially in private practice. To the peculiar restlessness and sleeplessness found in many insane persons, we have no doubt chloral is superior to opium or any of its preparations, and will soon be generally and habitually used in all that class of cases, and it may, to a too great extent, take the place of chloric ether, Hoffman's anodyne, hyoscyamus, and that class of remedies. We have no hope or fears of its ever taking the place of opium. That drug has not yet been discovered, and perhaps will never be, but we hope to live to see some new remedy or some new preparation of some old remedy, with all the sweet, soothing, happy and certain reliable effects of opium, without its harsh, sickening and injurious effects. For these new discoveries and improvements, the profession must look to the scientific and reliable pharmaceutist. The profession should know who they are, where they reside, and what they have already accomplished. It is a question, the importance of which has been overlooked,. that the profession concentrate its influence upon manufacturers and pharmaceutists who are reliable, and thus obtain pure drugs, and eradicate the numerous adulterations claiming to be pure drugs, from the land.

